# Binding proteins of destruxin A from *Metarhizium* against insect cell

**DOI:** 10.1186/s12866-023-02843-8

**Published:** 2023-04-04

**Authors:** Jingjing Wang, Qunfang Weng, Ke Zhang, Qiongbo Hu

**Affiliations:** 1 National Key Laboratory of Green Pesticide; Key Laboratory of Natural Pesticide and Chemical Biology, Ministry of Education, College of Plant Protection, South China Agricultural University, Guangzhou, 510642 China; 2 College of Horticulture, South China Agricultural University, Guangzhou, 510642 China

**Keywords:** Destruxin, Binding protein, Mode of action, *Metarhizium*

## Abstract

Destruxin A (DA) is a cyclo-hexadepsipeptidic insecticidal mycotoxin isolated from the entomopathogenic fungi, *Metarhizium* spp. However, its mode of action is unknown. In this study, we isolated 149 candidate DA-binding proteins by drug affinity response target stability, and determined the interactions of 80 canditates with DA in vitro by surface plasmon resonance. The affinity coefficients (K_D_) ranged from 24 to 469 μM. Binding proteins were functionally diverse and included cytoskeletal components and cell motility, protein transcription and translation pathways, ubiquitin dependent protein metabolic processes, nucleus pore entry and exit, and endoplasmic reticulum vesicle transport and etc. Electron microscopy revealed that DA damaged the cytoskeleton and multiple organelles, disrupted cell adhesion and motility, and led to cell death. DA appeared to have a multi-targeted approach to cellular structures and multiple life processes, leading to cell death. The results of this study could provide molecular evidence for the analysis of the insecticidal toxicology of DA and further improve the study of the pathogenic insect mechanism of *Metarhizium*.

**Supplementary Information:**

The online version contains supplementary material available at 10.1186/s12866-023-02843-8.

## Introduction

Pests seriously damage agriculture and forest. Pesticides play an important role in pest management of crop production. According to Insecticide Resistance Action Commetee, there are hundreds of insecticides, but there are only 34 MOAs (mode of action). Discovery of new MOAs is important mission for novel insecticides and pest resistant management. Elucidation of natural toxin’s MOA will absolutely promote novel pesticides. Destruxin A (DA), a mycotoxin of entomopathogenic fungi *Metarhizium* spp., is a non-ribosomal peptide with the hexa-cyclic peptide backbone [1–3]. DA has a high insecticidal activity against many insects, for example, the LD_50_ to Lepidopteran larvae is less than 0.1 μg/g by hemocoel injection or approximately 50 mg/L by oral or drip treatment [4, 5]. DA damages many tissues and cells in insects and affects the functions of the immune system, the muscular system and the Malpighian tubule [6–10].

Some researches suggested that DA is a cation ionophore to affect insect cell ion homeostasis [11, 12]. However, the effects of ion homeostasis need larger DA dosage (more than 100 mg/L), while lower dosage (less than 0.01 mg/L) has already alter the morphology of hemocytes and impair their immune function [8, 13]. More researches supported that DA is an insect innate immunosuppressant. It can cause abnormal expression of immune-related genes and proteins in insects. For example, the expression of some antimicrobial peptide genes is inhibited, while expression of genes such as phenoloxidase, serine proteases, and their inhibitors are induced [14–18]. Obviously, the current researches about the MOA of DA are ambiguous, it needs to further elucidated.

To explore the MOA of DA in this study, we isolated and identified DA binding proteins in Bm12 cell lines of the silkworm. We analyzed the molecular mechanism of DA causing cytotoxicity, and evaluated the results using electron microscopy for phenotype verification.

## Materials and methods

### Cell lines and compounds

The *Bombyx mori* Bm12 cell line was donated by Professor Cao Yang in South China Agricultural University. Cells were cultured in Grace’s insect medium with 10% fetal bovine serum. Cells were cultured in a constant-temperature incubator at 27℃. Logarithmic-phase cells were used for the experiment. All experimental cells were passed within 50 generations.

Destruxin A (DA) was isolated and purified *from Metarhizium anisopliae* strain MaQ10 in our laboratory [19, 20]. It was identified and quantified by HPLC and MS employing a standard of destruxin A bought from Sigma-Aldrich. Its purity was determined as more than 98%. It was dissolved using dimethyl sulfoxide as a stock of 10,000 mg/L and diluted with medium to the working concentration for the experiment.

### Drug affinity response target stability (DARTS)

In cell lysis group, total cellular protein was extracted using the animal cell total protein extraction kit. Simply, the sample was centrifuged at 12,000 rpm for 15 min, and the supernatant was dialyzed into TBS solution and quantified to 3000 μg/mL for enzymolysis. In live cells group, DA was added to Bm12 cells and incubated for 6 h, after which the protein was extracted and quantified for enzymolysis. Proteinase K solution (1 μg/mL) was added to the above protein, which was enzymatically digested for 1 min; and EDTA was added to inactivate the protease. Protein loading buffer was added into sample. Electrophoresis was performed after boiling water for 10 min. The difference bands were cut off and preserved, and the proteins were identified by mass spectrometry.

### Expression of candidate protein

The candidate gene was cloned by PCR, ligated into the expression vector pET28a, and transformed into BL21 cell for expression in the prokaryotic expression system. The protein was purified to a purity of 90% using Ni–NTA, and the affinity with DA was determined using SPR.

### Surface plasmon resonance (SPR)

The SPR expreiment was conducted by Biacore 8 K system. Firstly, the proteins were dialyzed into the PBS buffer at pH 7.4. Since the proteins were coupled to the chip by electrostatic interaction, the suitable pH needs to be figured out. The proteins were diluted using sodium acetate solutions of different pH values (4.0, 4.5, 5.0, 5.5), the protein concentration was diluted to about 0.3 mg/mL, and the pH values that coupled the most proteins was selected for the coupling experiment. According to the prompt of the instrument, EDC and NHS in the amino coupling kit were utilized to activate the chip, and the protein sample was injected after 5 min. After 5 min of injection, the chip was closed using the closure reagent to complete the protein coupling. Since DA was insoluble in water and required DMSO co-solubilization, the running buffer was configured as 1 × PBSP (5% DMSO), and successive gradients of DA solutions ( 62.5, 31.25, 15.63, 7.81, 3.90, 1.95 μM) were configured using the running buffer. In order to eliminate the interference of DMSO in the experiment, a corrective solution with 4.5%-5.8% DMSO content needed to be configured, with detailed steps are described in the Biacore 8 K user manual. After all the solutions are prepared, the experiment could be started by injecting and running the sample according to the instrument’s prompts. After the run, the kinetic constants of the DA and the suspected protein are calculated using the instrument’s own multi-cycle analysis method, and the experiment was repeated at least two times.

### SEM and TEM

A certain concentration of DA was added to Bm12 cells that were collected by centrifugation and preserved in fix solution (4% Glutaraldehyde and 2% Para-formaldehyde in 0.1 M phosphate buffer (PB), pH 7.0), and stored at 4℃. For SEM, the fixed samples were washed with PB for 4 times, and post-fixed with 1% osmium tetroxide (OsO4), followed by dehydration with gradient ethanol. Afterwards, the samples were dried with Critical Point Dryer. Specimens were attached to metallic stubs using carbon stickers and sputter-coated with gold for 30 s. Images were recorded and observed with a scanning electron microscope. For TEM, The fixed samples were washed with PB for 4 times, post-fixed with 1% OsO4, followed by dehydration with gradient ethanol. The samples were then transferred in acetone and embedded in Spurr’s resin and polymerized at 70℃ for 48 h. The sample blocks were cut into 70 nm sections by using an ultramicrotome (UC7, Leica). Sections were collected with copper grids and stained with uranyl acetate and lead citrate. The stained grids were inspected by Thermo Fisher Talos L120C electron microscope and and imaged by Ceta2 camera.

## Results

### Isolation of DA-binding proteins from Bm12 cells by drug affinity response target stability

We used the drug affinity response target stability (DARTS) method [21], which is based on the nature of proteins that are not susceptible to protease after binding to small molecules, to isolate DA-binding proteins in the Bm12 cell lines. Protein samples from the DA-treated group after protease treatment will have differential band compared with the control protein samples after electrophoresis. And the proteins in the differential bands can be identified as DA candidate binding proteins. This section is divided into two parts: (1) the cell lysis group, in which cell lysis was mixed with DA and then subjected to enzymolysis to directly investigate the binding protein of DA in Bm12 cells; (2) the DA treatment of live cells was evaluated, followed by total cellular protein extraction and then enzymolysis. These treatments allowed the binding proteins of DA in Bm12 cells to be studied. Furthermore, the effect of DA on the protein homeostasis of Bm12 cells was evaluated.

**Fig. 1:**
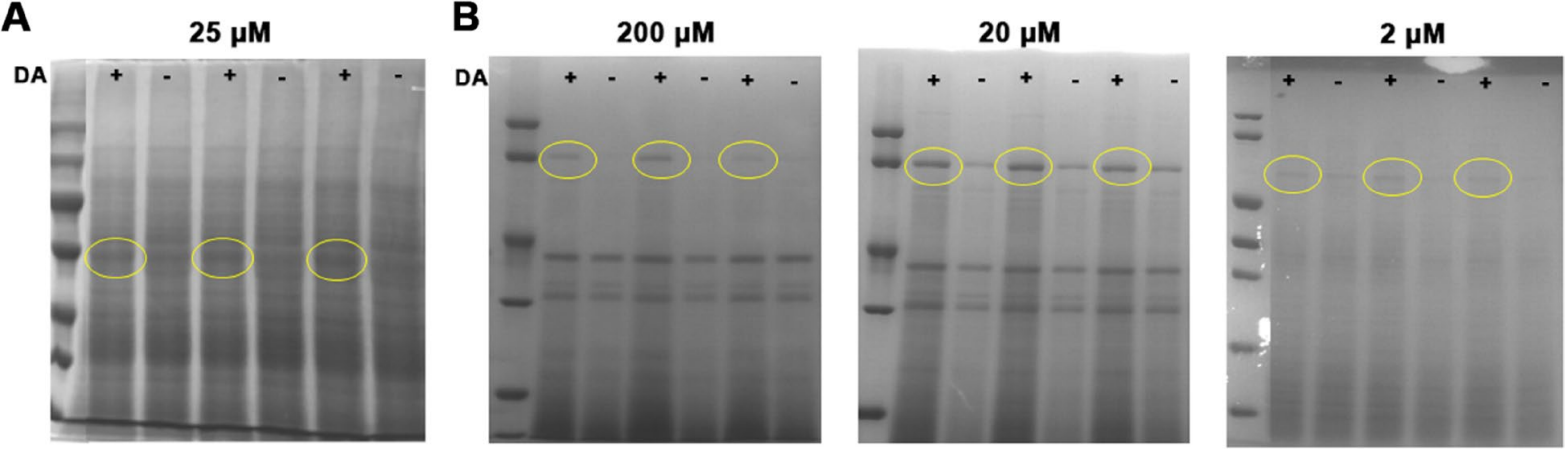
Isolation and identification of candidate DA-binding protein in Bm12 cell lines. **A** Isolation of candidate DA-binding protein in cell lysis group by DARTS. **B** Isolation of candidate DA-binding protein in live cell group by DARTS. Yellow circle: differential band; plus: DA treatment; minus: control group

After method development tests, proteinase K was selected for DARTS experiments, in which the protein concentration was about 3000 μg/mL, the proteinase K concentration was about 1 μg/mL and the digestion time was 1 min. Differential bands were found in the cell lysate group only at a DA dosage of 25 μM and three differential bands were collected for mass spectrometry (Fig. 1A). A total of 48 candidate binding proteins were identified (Table S1). Protein interaction analysis using the STRING database revealed that the candidates were not well associated with each other and could not form an interaction network. After clustering using the KEGG database, nearly half of the proteins were found to be clustered in genetic information processing and metabolic processes (Fig. S1). A total of three DA concentrations (2, 20 and 200 μM) were tested in the live cell group, and three differential bands were identified for each concentration (Fig. 1B). A total of 101 candidate binding proteins were identified (Table S2). No correlation was found between differential protein and DA treatment concentration. Analysis of protein interaction networks using the STRING database revealed that the most associated fraction of proteins were clustered in endoplasmic reticulum-associated, RNA transfer and amino acid tRNA synthase-associated pathways. The KEGG pathway clustering analysis of the proteins revealed that half of the proteins clustered in the genetic information processing pathway (Fig. S2).

### Identification of DA-binding protein by surface plasmon resonance

To verify whether the isolated candidate is a DA-binding protein, we expressed and purified the candidate proteins and determined the interaction of the candidate protein with DA in vitro using the surface plasmon resonance (SPR) method (Fig. S3). Some proteins were not expressed or could not be coupled to the SPR chip but we successfully determined the affinity of 80 candidates with DA (Fig. S4). The test proteins all interacted with DA with an affinity range of 24–469 μM (Table 1). These affinity represented moderate binding. And the affinity difference between each protein with DA was narrow. The DA-binding proteins were associated with a variety of cellular components and processes. For example, DA acted on cytoskeletal components and cell motility-related proteins, such as binding actin-4, myosin heavy chain, dynein heavy chain, alpha-actinin, alpha-tubulin, and microtubule-actin cross-linking factor 1. These proteins affected affecting cell morphology, motility and normal physiological functions. Furthermore, DA acted on protein synthesis pathway-related proteins, such as binding elongation factor 1 delta, EIF3-S8, elongation factor 1 alpha, eukaryotic translation initiation factor 3, translation elongation factor 2, and lysine tRNA ligase. These protein were involved with cellular protein synthesis, normal cellular metabolism and stress functions. DA also acted on ubiquitin-dependent proteins related to protein metabolic processes including the 26S protease regulatory subunit, proteasome 26S non-ATPase subunit, and ubiquitin carboxyl-terminal hydrolase 5, to disrupt the process of cellular degradation of proteins. DA bound to ATP synthase, calnexin and COP complex on the endoplasmic reticulum, and affected vesicle transport from the endoplasmic reticulum to the Golgi apparatus and protein fold. DA acted on importin and exportin on the nucleus pores, controlled the entry and exit of nuclear material and regulating cellular metabolism and genetics. As such, DA may use a multi-target strategy at a micromolar dosage level to affect cell function, and cause cell death.

**Table 1:** Assessing the interaction of DA with candidate proteins by surface plasmon resonance

Function or location	Protein annotation	K_D_ (μM)
Cytoskeleton and cell movement		
	Actin-4	25.6
	Laminin subunit gamma-1	28.6
	Myosin heavy chain, non-muscle	32.2
	Alpha-actinin	54.9
	CAD protein	38.8
	Dynein heavy chain, cytoplasmic	55.8
	Alpha-tubulin	69.5
	Microtubule-actin cross-linking factor 1	81.9
Translation		
	Elongation factor 1 delta	37.8
	EIF3-S8	45.0
	Elongation factor 1-alpha	61.1
	Eukaryotic translation initiation factor 3 subunit C isoform X1	82.8
	Translation elongation factor 2	85.9
	Lysine-tRNA ligase isoform X2	99.1
Ubiquitin–proteasome system		
	E3 UFM1-protein ligase 1 homolog	45.6
	26S protease regulatory subunit 6A-B	62.8
	Ubiquitin carboxyl-terminal hydrolase 5	93.4
	Phospholipase A-2-activating protein	95.6
	Ubiquitin-like modifier-activating enzyme 1	95.8
	Polyubiquitin	99.7
	Proteasome 26S non-ATPase subunit 12 isoform X1	264
Transcription		
	ATP-binding cassette sub-family F member 2 isoform X1	24.0
	Splicing factor proline- and glutamine-rich	46.6
	La-related protein 1	54.2
	Zinc finger CCCH domain-containing protein 15 homolog	65.0
	Poly A binding protein	65.6
	THO complex subunit 1	69.5
	mRNA-capping enzyme isoform X1	73.5
	Symplekin	79.0
	Leucine-rich PPR motif-containing protein	262
Nucleus		
	EN protein binding/engrailed nuclear homeoprotein-regulated protein	52.1
	Importin-5	35.1
	Importin-7 isoform X1	58.2
	Exportin-1	64.6
	Importin-7 isoform X2	99.6
	E3 SUMO-protein ligase RanBP2	96.5
	Nucleolar and coiled-body phosphoprotein 1	67.0
	Cell growth-regulating nucleolar protein	86.1
	Nucleolar protein 9	99.2
Endoplasmic reticulum		
	Transitional endoplasmic reticulum ATPase TER94	43.0
	Dolichyl-diphosphooligosaccharide protein glycotransferase	73.3
	COP complex beta subunit	60.9
	Calnexin	60.9
	Ribosome-binding protein 1 isoform X1	46.3
	Sarco/endoplasmic reticulum calcium ATPase	96.8
Peptidase		
	Prolyl oligopeptidase	27.0
	Protein disulfide isomerase precursor	44.6
	Cytosolic non-specific dipeptidase	59.3
	Xaa-Pro aminopeptidase 1	73.7
	Presequence protease, mitochondrial	81.2
Membrane protein		
	ATP synthase	40.3
	Limbic system-associated membrane protein	71.0
	H + transporting ATP synthase beta subunit isoform 2	84.0
	Beta-alanine transporter isoform X1	97.0
	Glycosylated lysosomal membrane protein	99.8
Others		
	Uncharacterized protein LOC101744639	99.4
	Uncharacterized protein LOC101744580	97.9
	Vacuolar protein sorting-associated protein 13C	95.2
	Muscle glycogen phosphorylase	91.7
	Lysosomal alpha-mannosidase-like_ partial	89.6
	Dicer-2 isoform X1	82.0
	Failed axon connections isoform X1	80.0
	Serrate RNA effector molecule homolog isoform X2	77.6
	U3 small nucleolar RNA-associated protein 14 homolog C	73.3
	Transketolase	72.9
	LETM1 and EF-hand domain-containing protein 1, mitochondrial	71.6
	Cytosolic purine 5'-nucleotidase isoform X2	71.2
	DnaJ-20	69.5
	Low-density lipoprotein receptor-related protein 2	68.5
	Dihydrolipoamide dehydrogenase	65.9
	Ras GTPase-activating protein-binding protein 2	62.7
	Neutral alpha-glucosidase AB-like precursor	61.9
	Uncharacterized protein LOC101735738 isoform X2	58.5
	Serine hydroxymethyltransferase	53.8
	Scavenger receptor class B member 3	50.1
	Uncharacterized protein LOC101735738 isoform X1	49.6
	Serine/threonine-protein phosphatase 2A	36.3
	Protein scabrous	469
	ATP-citrate synthase-like, partial	212
	ADP-ribosylation factor 2	205

### Cytotoxicity of DA against Bm12 cell lines

**Fig. 2:**
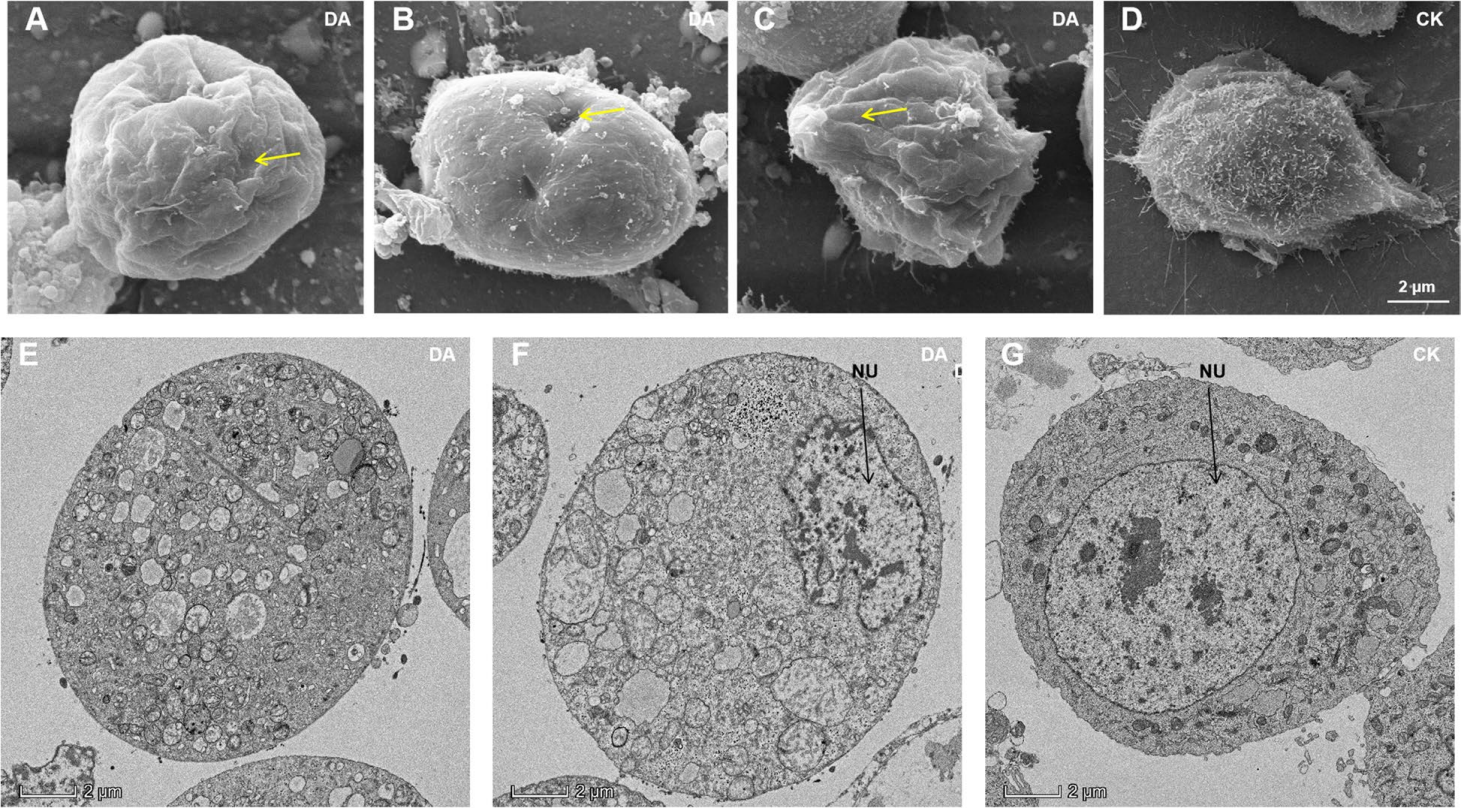
Electron microscopic results of the cytotoxicity of DA on Bm12. **A**, **B**, **C**, **D** Effects of DA on Bm12 cells morphology change by SEM. After DA treatment, the cell microvilli were significantly reduced (**A**, **B** and **C**), the cell morphology changed significantly, and the cell surface showed obvious shrinkages (**A** and **C**) and perforation (**B**). **E**, **F**, **G** Effects of DA on Bm12 cells by TEM. DA treated cells showed nuclear consolidation, cytoplasmic degradation, mitochondrial and endoplasmic reticulum swelling, and almost no intact organelles (**A** and **B**). NU: cell nucleus

We studied the cytotoxicity of DA on Bm12 cells by scanning and transmission electron microscopy. Based on the dose toxicity of DA on Bm12 cells, we used 50 μg/mL DA to treat the cells for 12 h. After treatment, the samples were prepared and observed. SEM results showed that after DA-treated, cells did not adhere to the wall, cell microvilli were significantly reduced, cell morphology was changed and cell surface showed shrinkage and perforation (Fig. 2A, B, C, D). The changes in cell morphology indicated that DA can act on the cytoskeleton, such as microtubules and microfilaments. Microfilaments are the main component of microvilli, and there were few microvilli in DA-treated cells. This result confirmed that DA inhibited the function of cytoskeletal and motility-related proteins by binding to them, and impairing cytoskeletal structure and motility or adhesion. In the TEM results, the DA-treated cells showed necrotic symptoms such as nuclear consolidation, cytoplasmic degradation, mitochondrial and endoplasmic reticulum swelling, and few intact organelles (Fig. 2E, F, G). These observation were consistent with the diverse functions of the identified DA-binding proteins. DA appeared to use a multi-target strategy and acted on a variety of cellular structures and proteins in biological processes to disrupt normal cellular physiological functions. Overall, the toxicity of DA to Bm12 cells was multifaceted and did not act exclusively on one organelle or induce death caused by a single mechanism.

## Discussion

We found that DA had good insecticidal activity and acted on many insect tissues and organs, but its insecticidal mode of action is unknown. The target protein of DA have not been identified in insects. To study the molecular toxicology of DA at the cellular level, we isolated and identified DA-binding proteins in Bm12 cell line. We isolated 149 candidate proteins and identified 80 binding proteins. The affinity range of DA and binding proteins ranged from 24–469 μM, which indicated moderate binding, and the affinity between each protein and DA interactions was narrow. The protein functions were diverse and included cytoskeleton components and cell motility-related proteins, protein transcription and translation pathway-related proteins and ubiquitin-dependent protein metabolic process-related proteins. Electron microscopy observations also revealed that DA damaged the cytoskeleton, disrupted cell adhesion and motility, damaged organelles, and led to cell death.

Previous studies have shown that DA acts on the hemocyte cytoskeleton to alter cell morphology and disrupt phagocytosis and encapsulation [17], and the present study, we found that DA acted on the Bm12 cytoskeleton to impair cell attachment and motility and reduce microvilli. The molecular mechanism of this phenomenon was caused by the interactions of DA with actin, alpha-actinin and alpha-tubulin, which are important proteins that comprise microtubules and microfilaments. DA also interacted with the myosin heavy chain, dynein heavy chain as well as microtubule-actin cross-linking factor 1, binding proteins of microtubules and microfilaments. Also, DA inhibited the function of binding proteins and may regulate the interactions between binding proteins, ultimately leading to impaired cytoskeleton functions. We previously demonstrated that DA interacted with seven amino acid tRNA synthetases to affect the efficiency of cellular protein synthesis [22, 23]. The role of this enzyme was to catalyze the formation of complexes between amino acids and the corresponding tRNAs, which played an important role in protein translation elongation. The present study also demonstrated that DA could interact with translation revelation and elongation factor components, such as elongation factor 1 delta, EIF3-S8, elongation factor 1-alpha, eukaryotic translation initiation factor 3, and translation elongation factor 2. This suggesting that DA was involved in regulating the regulation of cellular protein synthesis pathway. DA also acted on the ubiquitin proteasomal protein degradation pathway, inhibiting cellular protein degradation processes, affecting cellular degradation of erroneous proteins, and disrupting normal cellular physiological processes. We also found that DA acted on the ATP synthase, calnexin and COP complex on the endoplasmic reticulum and may affect the transport of vesicles from the endoplasmic reticulum to the Golgi apparatus and protein folding. We previously also found that DA could interact with another protein of this process, SEC23A [24]. Furthermore, DA interacted with some peptidases, such as prolyl oligopeptidase or protein disulfide isomerase precursor, which may be involved in the degradation of DA. This involved hydrolysis of DA to less toxic chain structures, or conversion of small molecule peptides to their own nutrients. Among them, the interaction of DA with Prolyl oligopeptidase may result from DA molecules containing proline structures that are specifically recognized and bound by Prolyl oligopeptidase. DA also regulateed the movement of substances in and out of the nucleus and even affected the transcription and regulation of genes. Overall, DA used a multi-target approach to effect several life processes and affected cell morphology and organelle structures as well as causing cell death.

The multi-target strategy of destruxin has resulted from the evolution of interactions between *Metarhizium* spp. and insects [25, 26]. When *Metarhizium* spp. invades a host insect, it secretes a variety of structurally similar destruxins that attack as many tissues and organs. These produce a diseased state, weakening insect resistance and enabling successfully fungus colonization. Destruxin has 39 homologs3, among which the more active ones are destruxin A and B. Their structures differ only in vinyl and ethyl, and DB is further oxidized to DA during the synthesis, but the molecular mechanisms of their actions differ. In hemocyte ATPase, the affinity of DB is 100 times higher than DA [13]. This subtle structural difference causes DB to have lower insecticidal activity than DA, but greater antitumor activity than DA. This multi-target strategy of DA may provide new directions for the development of novel insecticides. The cyclic peptide structure provides the backbone support for the molecular stability of DA, and vinyl, as the only active side chain of DA, is the structural guarantee for the multi-target strategy of DA. Vinyl has an electron-absorbing effect and easily forms hydrogen-bonding interactions with proteins. The advantage of multi-target drugs is that they are less prone to resistance and easier to manage resistance compared to single-target drugs. Single-target insecticides such as diamine insecticides bind to the Ca^2+^ channel of the ryanodine receptor and this mode of action is readily subject to resistance development [27].

## Supplementary Information

**Additional file 1:** **Table S1.** Candidate DA binding protein isolated in cell lysis group.

**Additional file 2:** **Table S2.** Candidate DA binding protein isolated in live cell group.

**Additional file 3:** **Figure S1.** Protein interaction analysis by STRING database (left) and KEGG pathway clustering analysis (right) in cell lysis group candidate proteins. **Figure S2.** Protein interaction analysis by STRING database (left) and KEGG pathway clustering analysis (right) in live cell group candidate proteins. **Figure S3.** Expression and purification of candidate protein. **Figure S4.** Detailed SPR results of interaction of DA with candidate proteins. 

## Data Availability

The datasets used and/or analysed during the current study available from the corresponding author on reasonable request.
