# Supplementary material for: Binding proteins of destruxin A from Metarhizium against insect cell
Source: BMC Microbiol. 2023 Apr 4;23:96. doi: 10.1186/s12866-023-02843-8 (PMC10071776; doi:10.1186/s12866-023-02843-8)

Figure S1. Protein interaction analysis by STRING database (left) and KEGG pathway clustering analysis (right) in cell lysis group candidate proteins.

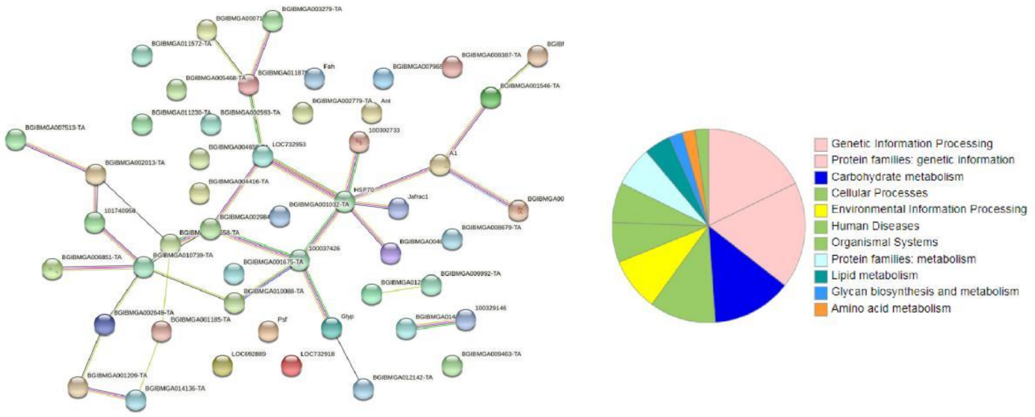

Figure S2. Protein interaction analysis by STRING database (left) and KEGG pathway clustering analysis (right) in live cell group candidate proteins.

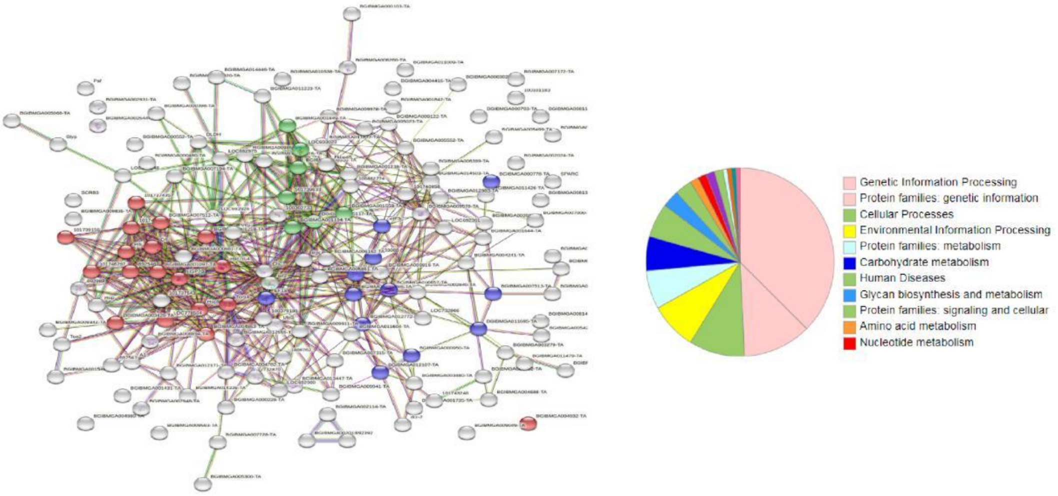

Figure S3. Expression and purification of candidate protein.

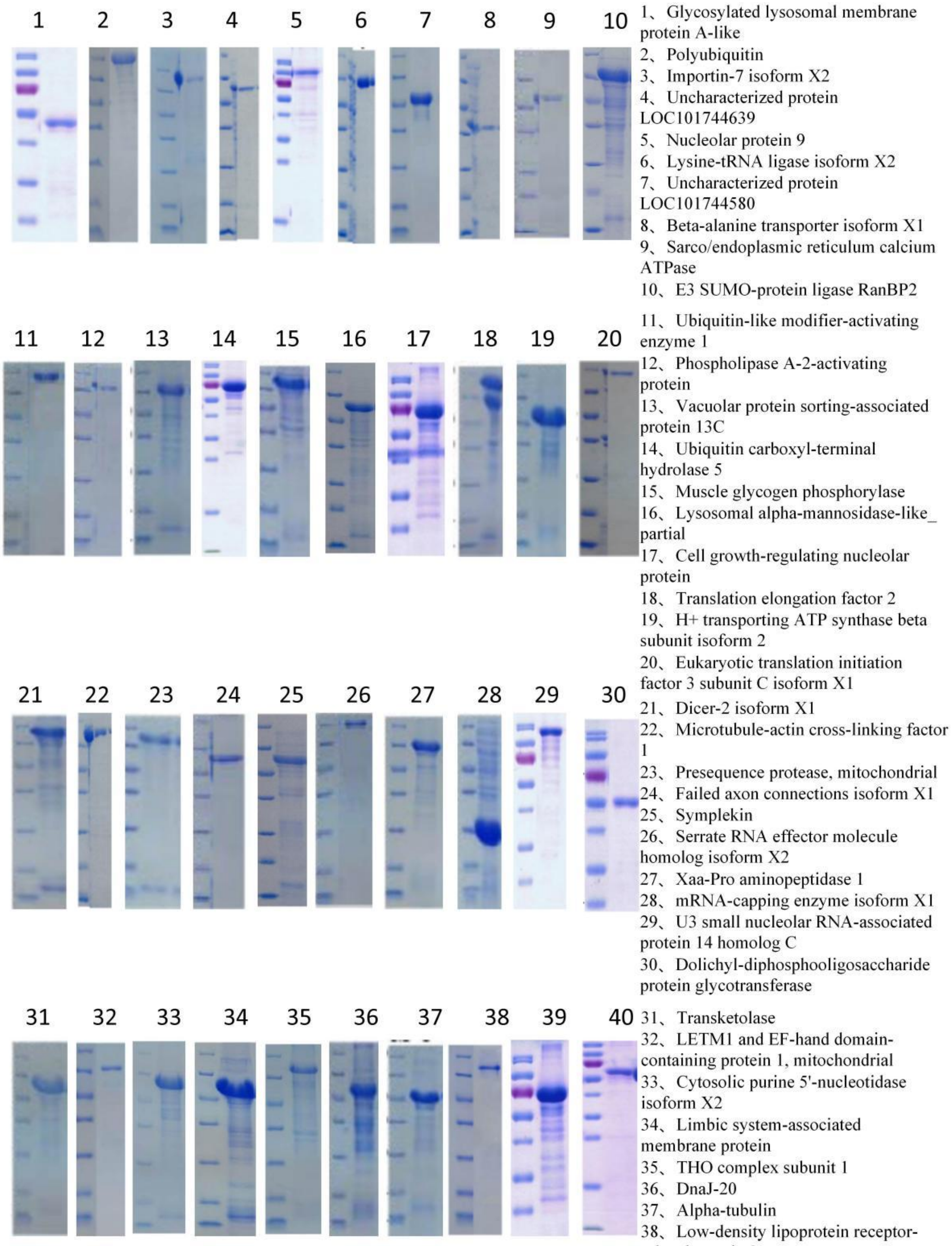

1、Glycosylated lysosomal membrane protein A-like
2、Polyubiquitin
3、Importin-7 isoform X2
4、Uncharacterized protein LOC101744639
5、Nucleolar protein 9
6、Lysine-tRNA ligase isoform X2
7、Uncharacterized protein LOC101744580
8、Beta-alanine transporter isoform X1
9、Sarco/endoplasmic reticulum calcium ATPase
10、E3 SUMO-protein ligase RanBP2
11、Ubiquitin-like modifier-activating enzyme 1
12、Phospholipase A-2-activating protein
13、Vacuolar protein sorting-associated protein 13C
14、Ubiquitin carboxyl-terminal hydrolase 5
15、Muscle glycogen phosphorylase
16、Lysosomal alpha-mannosidase-like_ partial
17、Cell growth-regulating nucleolar protein
18、Translation elongation factor 2
19、H+ transporting ATP synthase beta subunit isoform 2
20、Eukaryotic translation initiation factor 3 subunit C isoform X1
21、Dicer-2 isoform X1
22、Microtubule-actin cross-linking factor 1
23、Presequence protease, mitochondrial
24、Failed axon connections isoform X1
25、Symplekin
26、Serrate RNA effector molecule homolog isoform X2
27、Xaa-Pro aminopeptidase 1
28、mRNA-capping enzyme isoform X1
29、U3 small nucleolar RNA-associated protein 14 homolog C
30、Dolichyl-diphosphooligosaccharide protein glycotransferase
31、Transketolase
32、LETM1 and EF-hand domain-containing protein 1, mitochondrial
33、Cytosolic purine 5'-nucleotidase isoform X2
34、Limbic system-associated membrane protein
35、THO complex subunit 1
36、DnaJ-20
37、Alpha-tubulin
38、Low-density lipoprotein receptor-related protein 2
39、Nucleolar and coiled-body phosphoprotein 1
40、Dihydrolipoamide dehydrogenase

Continued

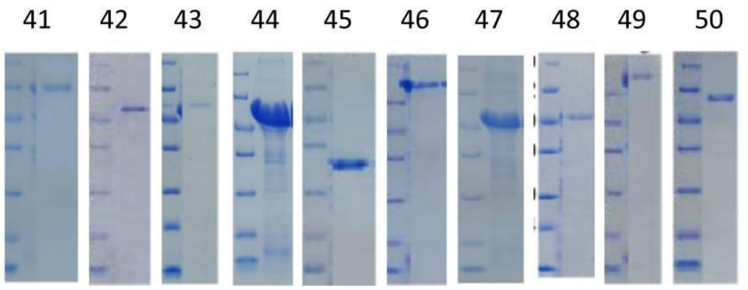

41、Poly A binding protein
42、Zinc finger CCCH domain-containing protein 15 homolog
43、Exportin-1
44、26S protease regulatory subunit 6A-B
45、Ras GTPase-activating protein-binding protein 2
46、Neutral alpha-glucosidase AB-like precursor
47、Elongation factor 1-alpha
48、Calnexin
49、COP complex beta subunit
50、Cytosolic non-specific dipeptidase

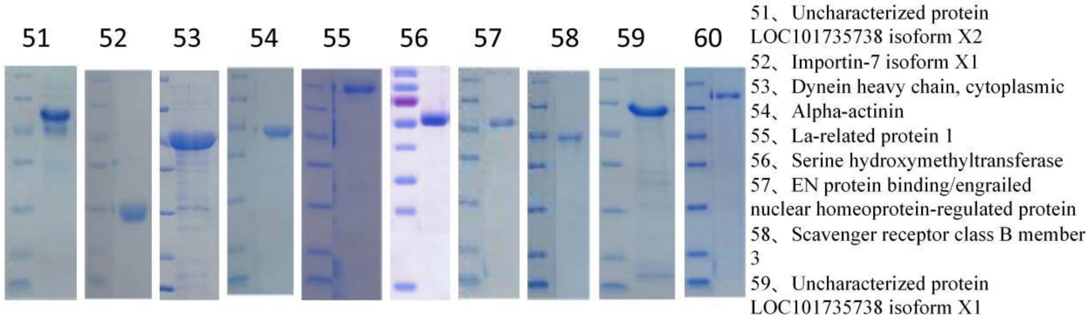

51、Uncharacterized protein LOC101735738 isoform X2
52、Importin-7 isoform X1
53、Dynein heavy chain, cytoplasmic
54、Alpha-actinin
55、La-related protein 1
56、Serine hydroxymethyltransferase
57、EN protein binding/engrailed nuclear homeoprotein-regulated protein
58、Scavenger receptor class B member 3
59、Uncharacterized protein LOC101735738 isoform X1
60、Splicing factor proline- and glutamine-rich

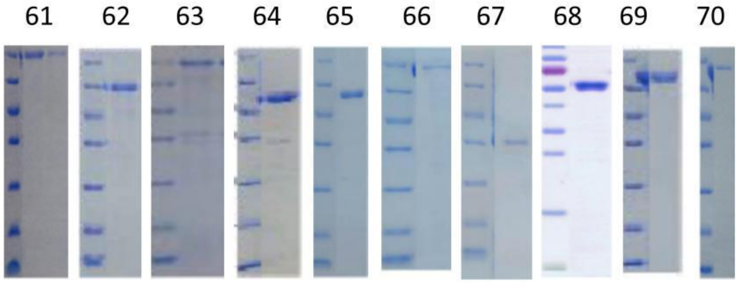

61、Ribosome-binding protein 1 isoform X1
62、E3 UFM1-protein ligase 1 homolog
63、EIF3-S8
64、Protein disulfide isomerase precursor
65、ATP synthase
66、CAD protein
67、Elongation factor 1 delta
68、Serine/threonine-protein phosphatase 2A
69、Importin-5
70、Myosin heavy chain, non-muscle

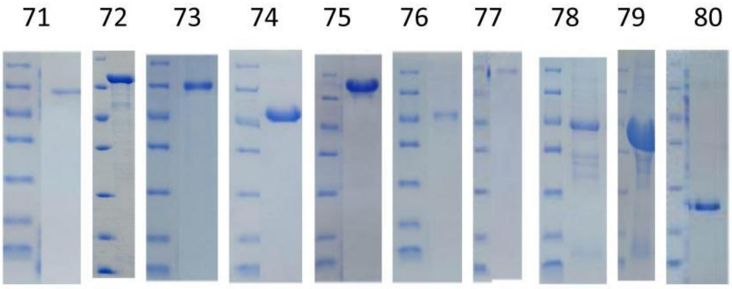

71、Laminin subunit gamma-1
72、Prolyl oligopeptidase
73、ATP-binding cassette sub-family F member 2 isoform X1
74、Protein scabrous
75、Transitional endoplasmic reticulum ATPase TER94
76、Proteasome 26S non-ATPase subunit 12 isoform X1
77、Leucine-rich PPR motif-containing protein
78、Actin-4
79、ATP-citrate synthase-like, partial
80、ADP-ribosylation factor 2

Figure S4. Detailed SPR results of interaction of DA with candidate proteins.

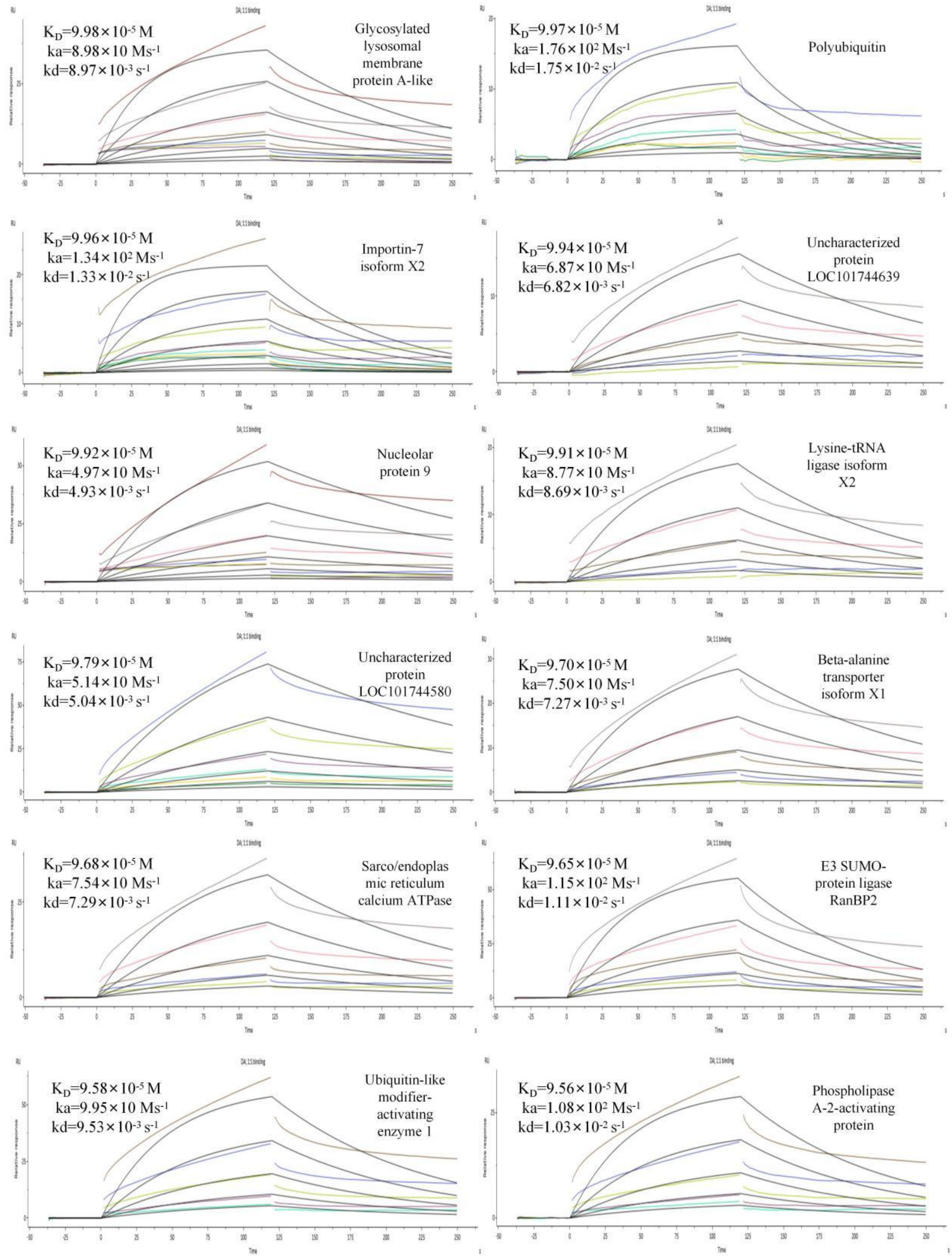

Continued

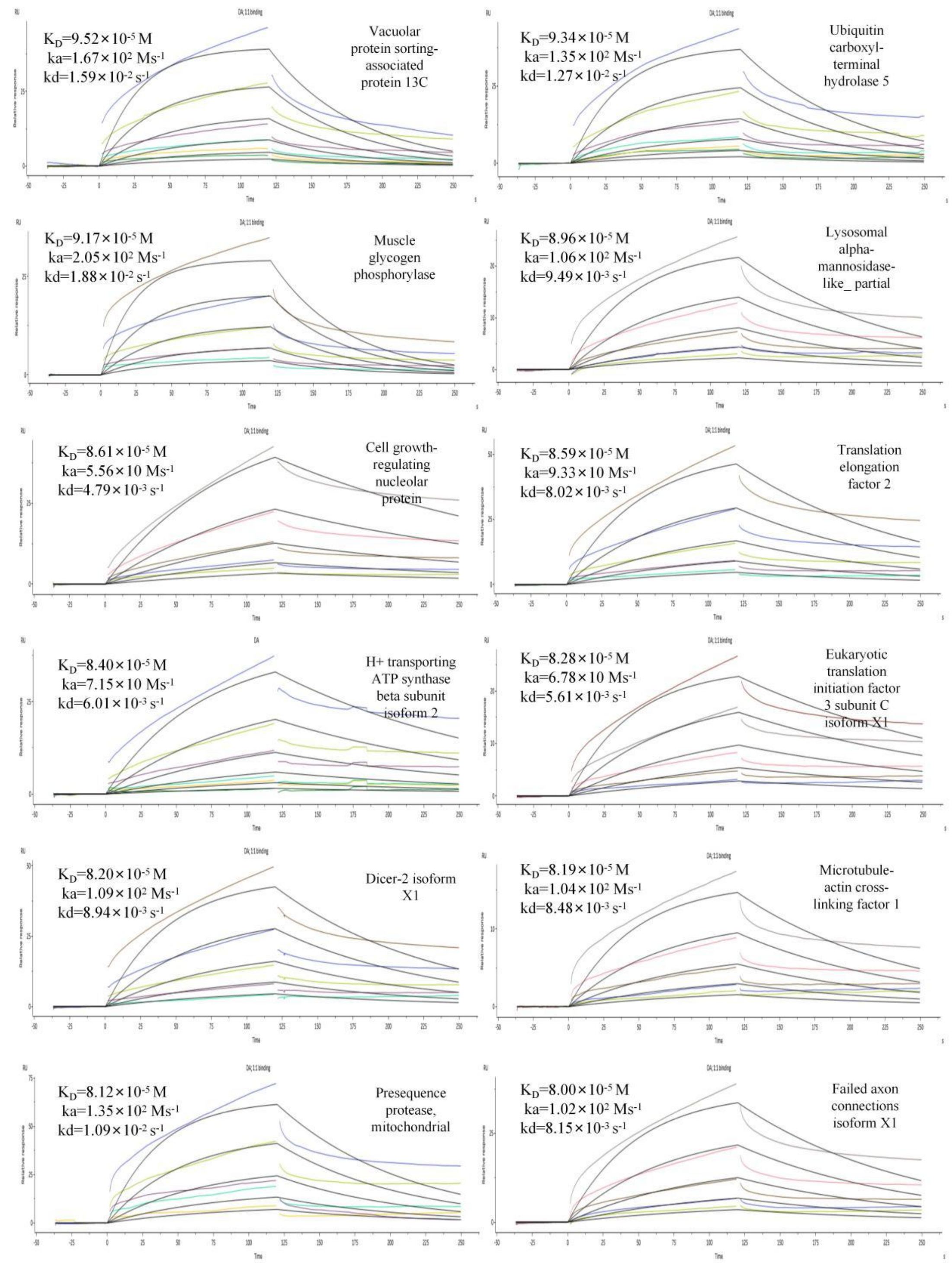
K_D=9.52×10^{-5} M
ka=1.67×10^{2} Ms^{-1}
kd=1.59×10^{-2} s^{-1}
Vacuolar protein sorting-associated protein 13C
K_D=9.34×10^{-5} M
ka=1.35×10^{2} Ms^{-1}
kd=1.27×10^{-2} s^{-1}
Ubiquitin carboxyl-terminal hydrolase 5
K_D=9.17×10^{-5} M
ka=2.05×10^{2} Ms^{-1}
kd=1.88×10^{-2} s^{-1}
Muscle glycogen phosphorylase
K_D=8.96×10^{-5} M
ka=1.06×10^{2} Ms^{-1}
kd=9.49×10^{-3} s^{-1}
Lysosomal alpha-mannosidase-like_ partial
K_D=8.61×10^{-5} M
ka=5.56×10 Ms^{-1}
kd=4.79×10^{-3} s^{-1}
Cell growth-regulating nucleolar protein
K_D=8.59×10^{-5} M
ka=9.33×10 Ms^{-1}
kd=8.02×10^{-3} s^{-1}
Translation elongation factor 2
K_D=8.40×10^{-5} M
ka=7.15×10 Ms^{-1}
kd=6.01×10^{-3} s^{-1}
H+ transporting ATP synthase beta subunit isoform 2
K_D=8.28×10^{-5} M
ka=6.78×10 Ms^{-1}
kd=5.61×10^{-3} s^{-1}
Eukaryotic translation initiation factor 3 subunit C isoform X1
K_D=8.20×10^{-5} M
ka=1.09×10^{2} Ms^{-1}
kd=8.94×10^{-3} s^{-1}
Dicer-2 isoform X1
K_D=8.19×10^{-5} M
ka=1.04×10^{2} Ms^{-1}
kd=8.48×10^{-3} s^{-1}
Microtubule-actin cross-linking factor 1
K_D=8.12×10^{-5} M
ka=1.35×10^{2} Ms^{-1}
kd=1.09×10^{-2} s^{-1}
Presequence protease, mitochondrial
K_D=8.00×10^{-5} M
ka=1.02×10^{2} Ms^{-1}
kd=8.15×10^{-3} s^{-1}
Failed axon connections isoform X1

Continued

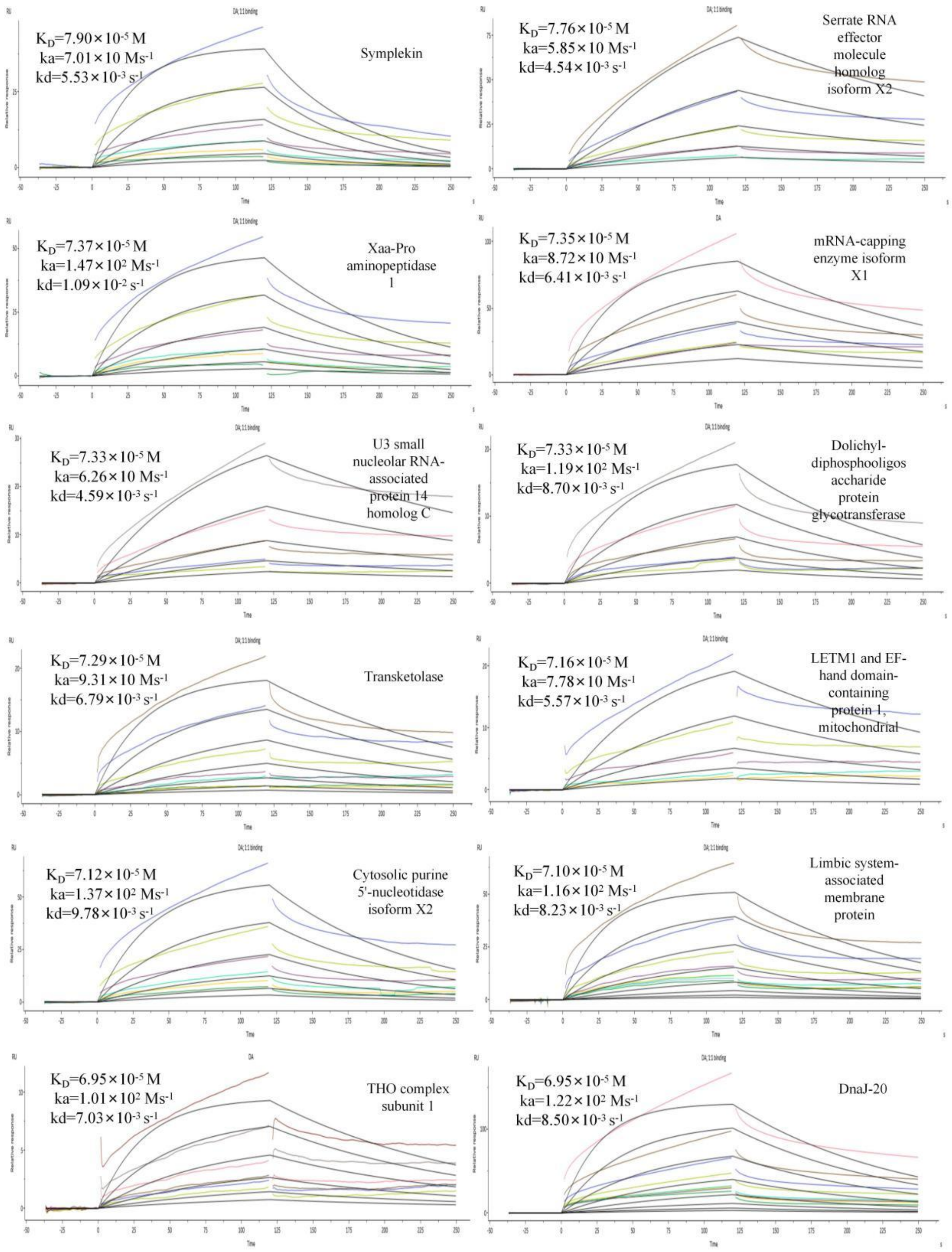

KD=7.90×10-5 M
ka=7.01×10 Ms-1
kd=5.53×10-3 s-1
Symplekin
KD=7.76×10-5 M
ka=5.85×10 Ms-1
kd=4.54×10-3 s-1
Serrate RNA effector molecule homolog isoform X2
KD=7.37×10-5 M
ka=1.47×102 Ms-1
kd=1.09×10-2 s-1
Xaa-Pro aminopeptidase 1
KD=7.35×10-5 M
ka=8.72×10 Ms-1
kd=6.41×10-3 s-1
mRNA-capping enzyme isoform X1
KD=7.33×10-5 M
ka=6.26×10 Ms-1
kd=4.59×10-3 s-1
U3 small nucleolar RNA-associated protein 14 homolog C
KD=7.33×10-5 M
ka=1.19×102 Ms-1
kd=8.70×10-3 s-1
Dolichyl-diphosphooligos accharide protein glycotransferase
KD=7.29×10-5 M
ka=9.31×10 Ms-1
kd=6.79×10-3 s-1
Transketolase
KD=7.16×10-5 M
ka=7.78×10 Ms-1
kd=5.57×10-3 s-1
LETM1 and EF-hand domain-containing protein 1, mitochondrial
KD=7.12×10-5 M
ka=1.37×102 Ms-1
kd=9.78×10-3 s-1
Cytosolic purine 5'-nucleotidase isoform X2
KD=7.10×10-5 M
ka=1.16×102 Ms-1
kd=8.23×10-3 s-1
Limbic system-associated membrane protein
KD=6.95×10-5 M
ka=1.01×102 Ms-1
kd=7.03×10-3 s-1
THO complex subunit 1
KD=6.95×10-5 M
ka=1.22×102 Ms-1
kd=8.50×10-3 s-1
DnaJ-20

Continued

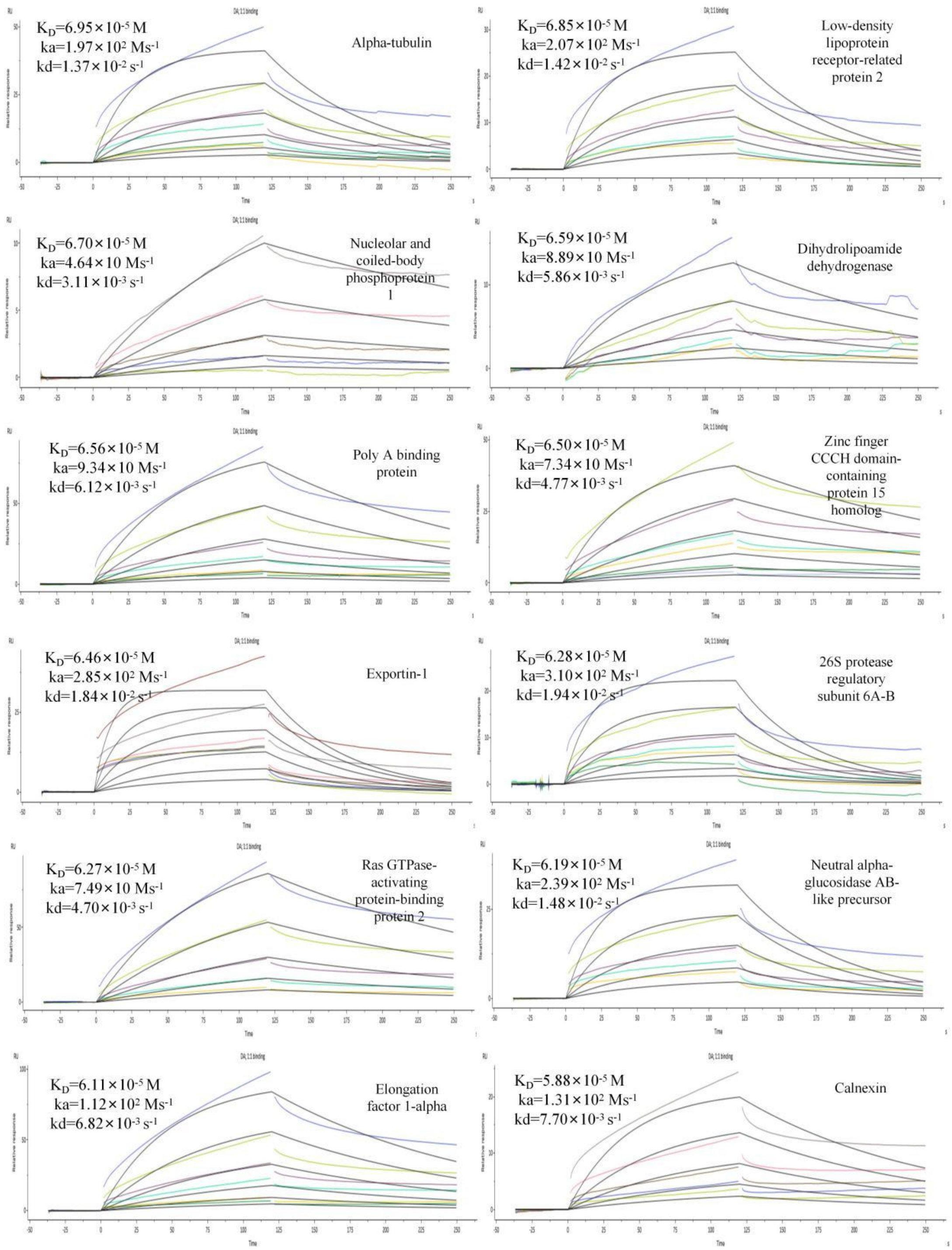
K_D=6.95×10^{-5} M
ka=1.97×10^{2} Ms^{-1}
kd=1.37×10^{-2} s^{-1}
Alpha-tubulin
K_D=6.85×10^{-5} M
ka=2.07×10^{2} Ms^{-1}
kd=1.42×10^{-2} s^{-1}
Low-density lipoprotein receptor-related protein 2
K_D=6.70×10^{-5} M
ka=4.64×10 Ms^{-1}
kd=3.11×10^{-3} s^{-1}
Nucleolar and coiled-body phosphoprotein 1
K_D=6.59×10^{-5} M
ka=8.89×10 Ms^{-1}
kd=5.86×10^{-3} s^{-1}
Dihydrolipoamide dehydrogenase
K_D=6.56×10^{-5} M
ka=9.34×10 Ms^{-1}
kd=6.12×10^{-3} s^{-1}
Poly A binding protein
K_D=6.50×10^{-5} M
ka=7.34×10 Ms^{-1}
kd=4.77×10^{-3} s^{-1}
Zinc finger CCCH domain-containing protein 15 homolog
K_D=6.46×10^{-5} M
ka=2.85×10^{2} Ms^{-1}
kd=1.84×10^{-2} s^{-1}
Exportin-1
K_D=6.28×10^{-5} M
ka=3.10×10^{2} Ms^{-1}
kd=1.94×10^{-2} s^{-1}
26S protease regulatory subunit 6A-B
K_D=6.27×10^{-5} M
ka=7.49×10 Ms^{-1}
kd=4.70×10^{-3} s^{-1}
Ras GTPase-activating protein-binding protein 2
K_D=6.19×10^{-5} M
ka=2.39×10^{2} Ms^{-1}
kd=1.48×10^{-2} s^{-1}
Neutral alpha-glucosidase AB-like precursor
K_D=6.11×10^{-5} M
ka=1.12×10^{2} Ms^{-1}
kd=6.82×10^{-3} s^{-1}
Elongation factor 1-alpha
K_D=5.88×10^{-5} M
ka=1.31×10^{2} Ms^{-1}
kd=7.70×10^{-3} s^{-1}
Calnexin

Continued

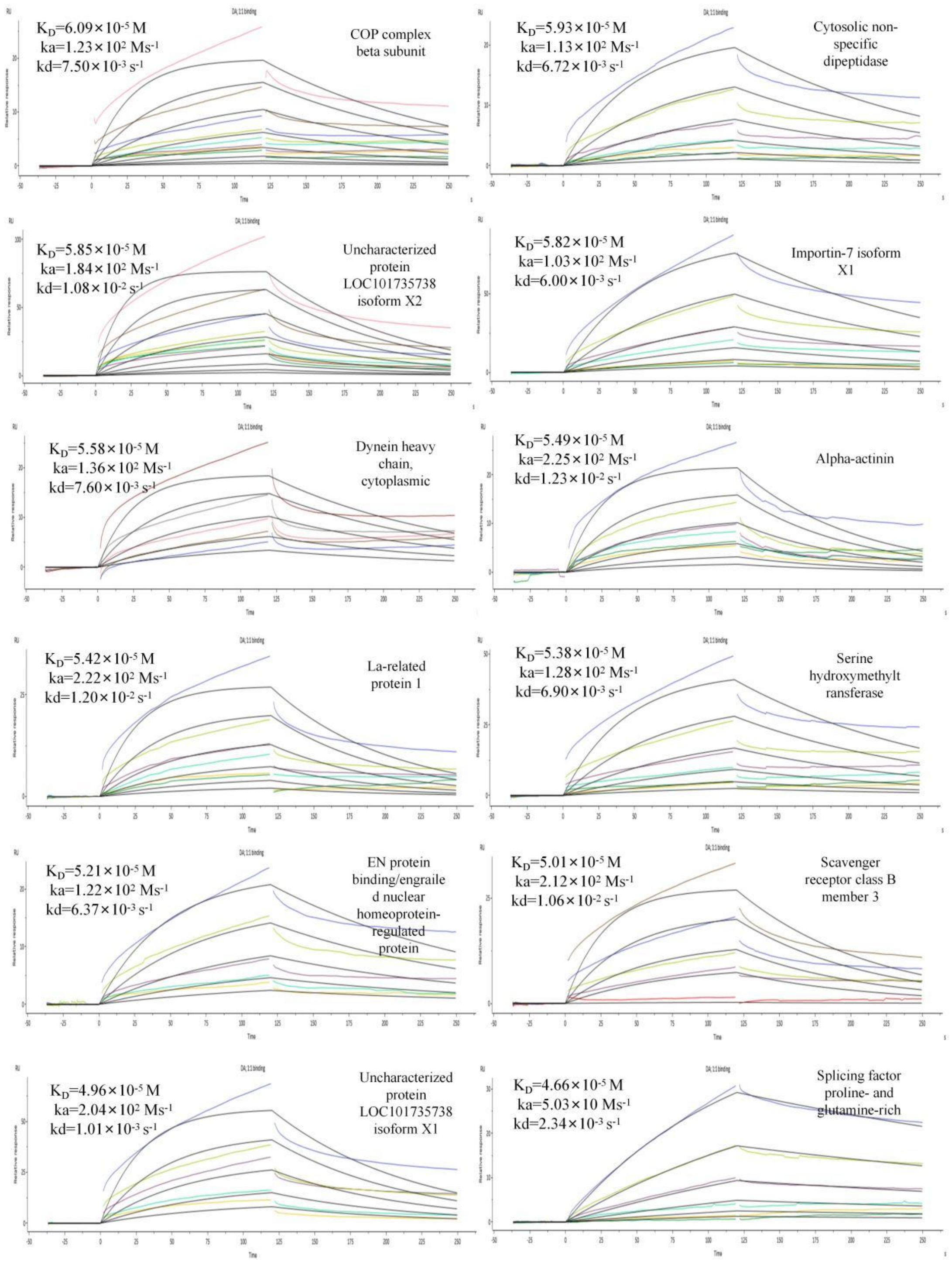
K_D=6.09×10^{-5} M
ka=1.23×10^{2} Ms^{-1}
kd=7.50×10^{-3} s^{-1}
COP complex beta subunit
K_D=5.93×10^{-5} M
ka=1.13×10^{2} Ms^{-1}
kd=6.72×10^{-3} s^{-1}
Cytosolic non-specific dipeptidase
K_D=5.85×10^{-5} M
ka=1.84×10^{2} Ms^{-1}
kd=1.08×10^{-2} s^{-1}
Uncharacterized protein LOC101735738 isoform X2
K_D=5.82×10^{-5} M
ka=1.03×10^{2} Ms^{-1}
kd=6.00×10^{-3} s^{-1}
Importin-7 isoform X1
K_D=5.58×10^{-5} M
ka=1.36×10^{2} Ms^{-1}
kd=7.60×10^{-3} s^{-1}
Dynein heavy chain, cytoplasmic
K_D=5.49×10^{-5} M
ka=2.25×10^{2} Ms^{-1}
kd=1.23×10^{-2} s^{-1}
Alpha-actinin
K_D=5.42×10^{-5} M
ka=2.22×10^{2} Ms^{-1}
kd=1.20×10^{-2} s^{-1}
La-related protein 1
K_D=5.38×10^{-5} M
ka=1.28×10^{2} Ms^{-1}
kd=6.90×10^{-3} s^{-1}
Serine hydroxymethyltransferase
K_D=5.21×10^{-5} M
ka=1.22×10^{2} Ms^{-1}
kd=6.37×10^{-3} s^{-1}
EN protein binding/engrailed nuclear homeoprotein-regulated protein
K_D=5.01×10^{-5} M
ka=2.12×10^{2} Ms^{-1}
kd=1.06×10^{-2} s^{-1}
Scavenger receptor class B member 3
K_D=4.96×10^{-5} M
ka=2.04×10^{2} Ms^{-1}
kd=1.01×10^{-3} s^{-1}
Uncharacterized protein LOC101735738 isoform X1
K_D=4.66×10^{-5} M
ka=5.03×10 Ms^{-1}
kd=2.34×10^{-3} s^{-1}
Splicing factor proline- and glutamine-rich
Relative response
Time

Continued

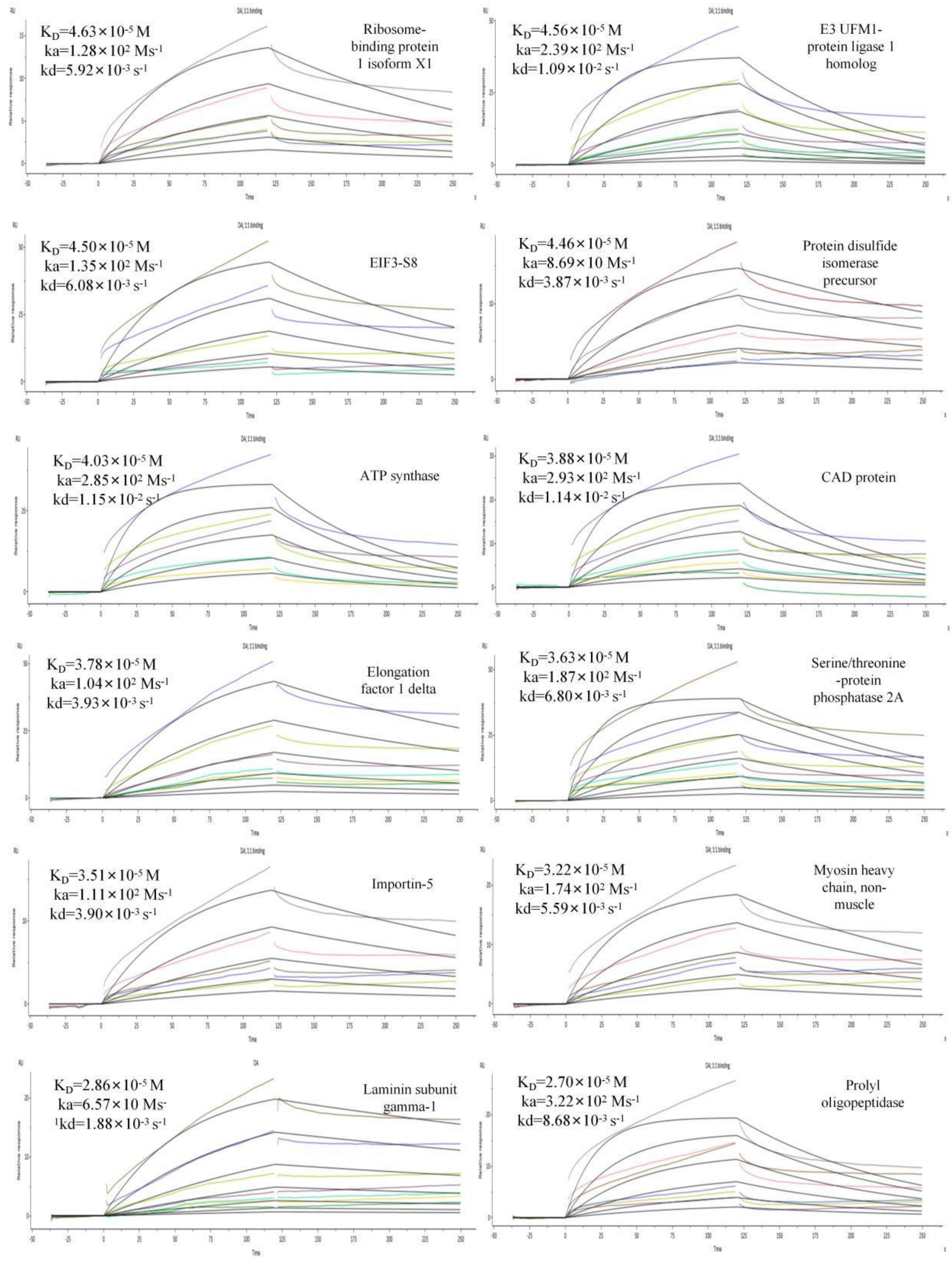

K_D=4.63×10^{-5} M
ka=1.28×10^{2} Ms^{-1}
kd=5.92×10^{-3} s^{-1}
Ribosome-binding protein 1 isoform X1
K_D=4.56×10^{-5} M
ka=2.39×10^{2} Ms^{-1}
kd=1.09×10^{-2} s^{-1}
E3 UFM1-protein ligase 1 homolog
K_D=4.50×10^{-5} M
ka=1.35×10^{2} Ms^{-1}
kd=6.08×10^{-3} s^{-1}
EIF3-S8
K_D=4.46×10^{-5} M
ka=8.69×10 Ms^{-1}
kd=3.87×10^{-3} s^{-1}
Protein disulfide isomerase precursor
K_D=4.03×10^{-5} M
ka=2.85×10^{2} Ms^{-1}
kd=1.15×10^{-2} s^{-1}
ATP synthase
K_D=3.88×10^{-5} M
ka=2.93×10^{2} Ms^{-1}
kd=1.14×10^{-2} s^{-1}
CAD protein
K_D=3.78×10^{-5} M
ka=1.04×10^{2} Ms^{-1}
kd=3.93×10^{-3} s^{-1}
Elongation factor 1 delta
K_D=3.63×10^{-5} M
ka=1.87×10^{2} Ms^{-1}
kd=6.80×10^{-3} s^{-1}
Serine/threonine-protein phosphatase 2A
K_D=3.51×10^{-5} M
ka=1.11×10^{2} Ms^{-1}
kd=3.90×10^{-3} s^{-1}
Importin-5
K_D=3.22×10^{-5} M
ka=1.74×10^{2} Ms^{-1}
kd=5.59×10^{-3} s^{-1}
Myosin heavy chain, non-muscle
K_D=2.86×10^{-5} M
ka=6.57×10 Ms^{-1}
kd=1.88×10^{-3} s^{-1}
Laminin subunit gamma-1
K_D=2.70×10^{-5} M
ka=3.22×10^{2} Ms^{-1}
kd=8.68×10^{-3} s^{-1}
Prolyl oligopeptidase

Continued

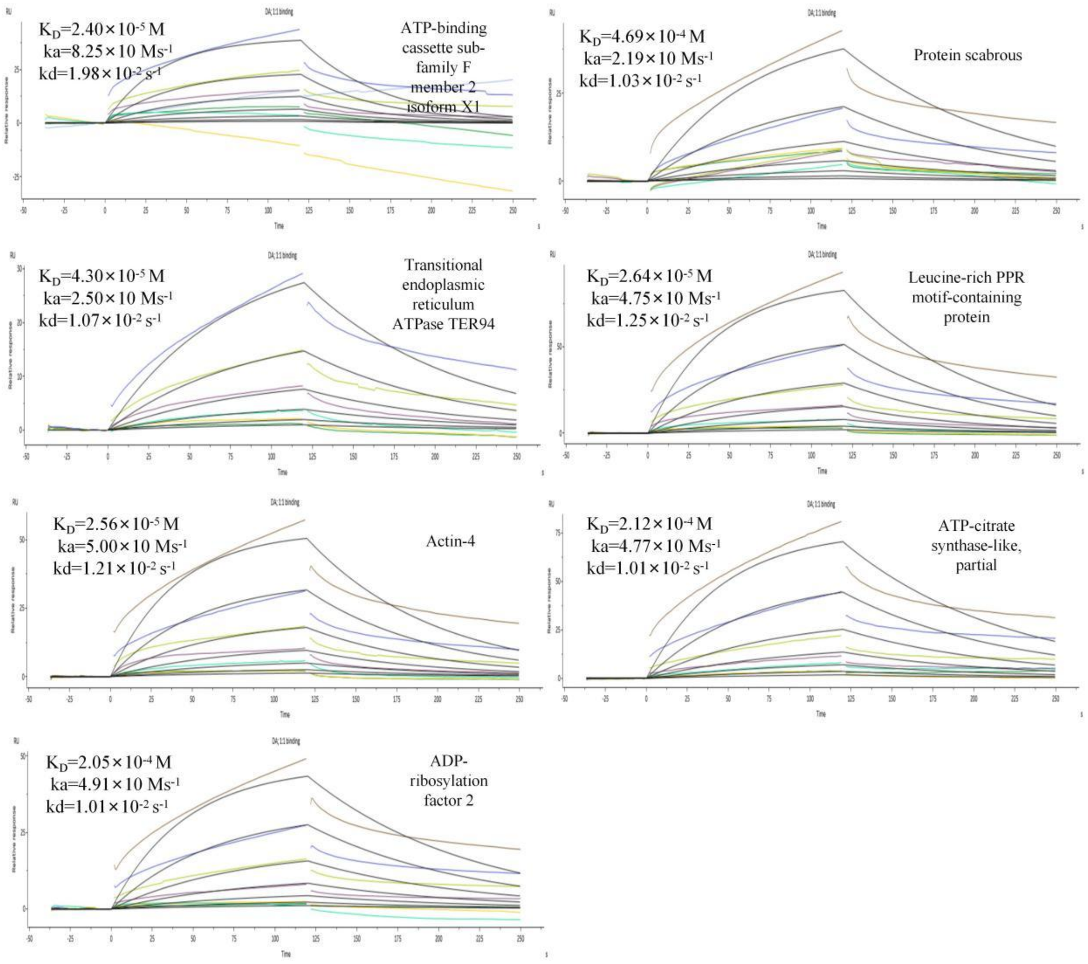
K_D=2.40×10^{-5} M
ka=8.25×10 Ms^{-1}
kd=1.98×10^{-2} s^{-1}
ATP-binding cassette sub-family F member 2 isoform X1
K_D=4.69×10^{-4} M
ka=2.19×10 Ms^{-1}
kd=1.03×10^{-2} s^{-1}
Protein scabrous
K_D=4.30×10^{-5} M
ka=2.50×10 Ms^{-1}
kd=1.07×10^{-2} s^{-1}
Transitional endoplasmic reticulum ATPase TER94
K_D=2.64×10^{-5} M
ka=4.75×10 Ms^{-1}
kd=1.25×10^{-2} s^{-1}
Leucine-rich PPR motif-containing protein
K_D=2.56×10^{-5} M
ka=5.00×10 Ms^{-1}
kd=1.21×10^{-2} s^{-1}
Actin-4
K_D=2.12×10^{-4} M
ka=4.77×10 Ms^{-1}
kd=1.01×10^{-2} s^{-1}
ATP-citrate synthase-like, partial
K_D=2.05×10^{-4} M
ka=4.91×10 Ms^{-1}
kd=1.01×10^{-2} s^{-1}
ADP-ribosylation factor 2

Original images

Figure 1A

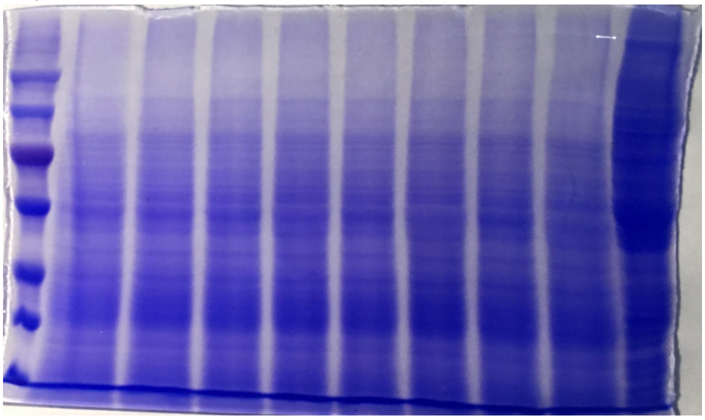

Figure 1B

200 µM

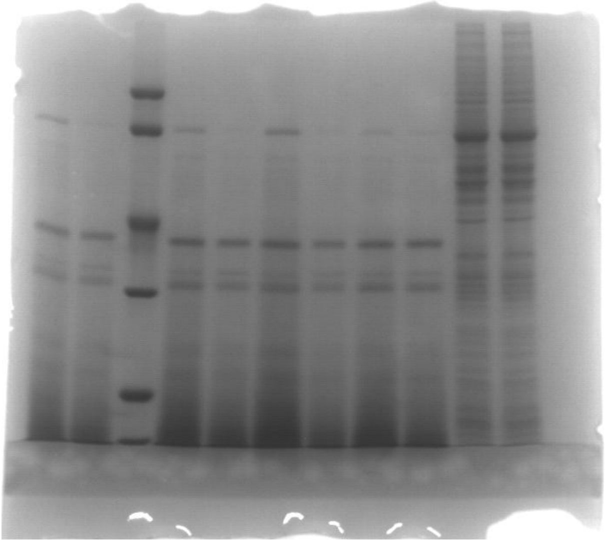

20 µM

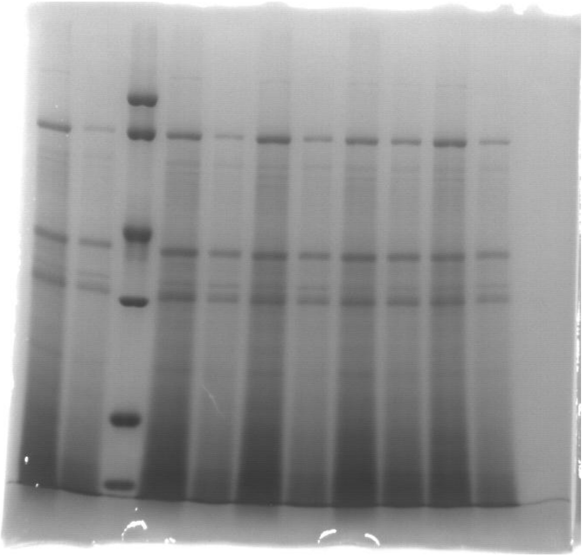

2 μM

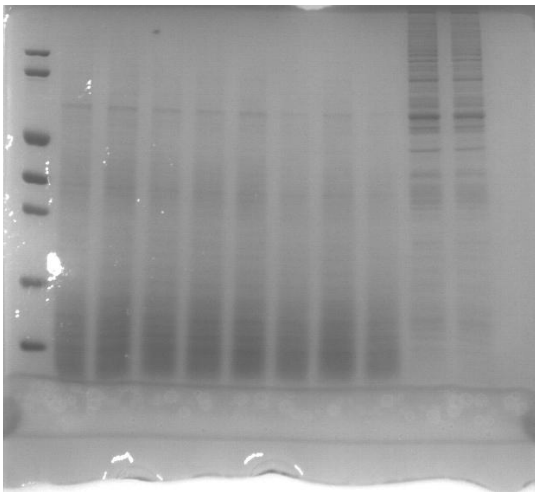

Supplement: Supplementary file 3 — Additional file 3: Figure S1. Protein interaction analysis by STRING database (left) and KEGG pathway clustering analysis (right) in cell lysis group candidate proteins. Figure S2. Protein interaction analysis by STRING database (left) and KEGG pathway clustering analysis (right) in live cell group candidate proteins. Figure S3. Expression and purification of candidate protein. Figure S4. Detailed SPR results of interaction of DA with candidate proteins. [file 12866_2023_2843_MOESM3_ESM.pdf]
